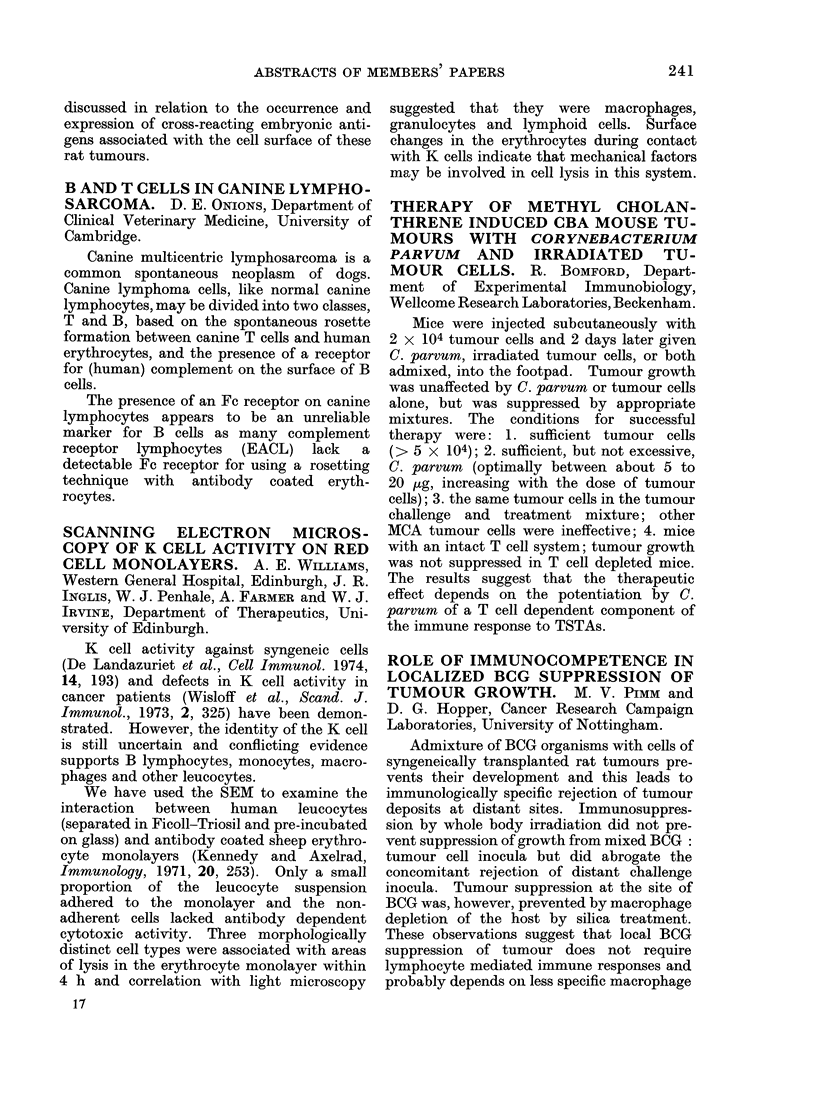# Proceedings: B and T cells in canine lymphosarcoma.

**DOI:** 10.1038/bjc.1975.157

**Published:** 1975-08

**Authors:** D. E. Onions


**B AND T CELLS IN CANINE LYMPHOSARCOMA.** D. E. Onions, Department of Clinical Veterinary Medicine, University of Cambridge.

Canine multicentric lymphosarcoma is a common spontaneous neoplasm of dogs. Canine lymphoma cells, like normal canine lymphocytes, may be divided into two classes, T and B, based on the spontaneous rosette formation between canine T cells and human erythrocytes, and the presence of a receptor for (human) complement on the surface of B cells.

The presence of an Fc receptor on canine lymphocytes appears to be an unreliable marker for B cells as many complement receptor lymphocytes (EACL) lack a detectable Fc receptor for using a rosetting technique with antibody coated erythrocytes.